# Impact of SARS-CoV-2 Infection on Pulmonary Function in the PURE-Colombia Cohort: A Comparative Analysis with Pre-COVID Values and Non-COVID-19 Controls

**DOI:** 10.3390/jcm15051868

**Published:** 2026-02-28

**Authors:** Heiler Lozada-Ramos, Ruth Aralí Martínez-Vega, Maritza Pérez-Mayorga, Patricio López-Jaramillo, Sumathy Rangarajan, MyLinh Duong, Salim Yusuf, Darryl Leong, Liliana Torcoroma García Sánchez

**Affiliations:** 1 Infectious Disease Research Program, Universidad de Santander (UDES), Bucaramanga 680006, Colombia; 2 Medicine Program, Universidad de Santiago de Cali, Santiago de Cali 760001, Colombia; 3 Health and Movement Research Group, Universidad de Santiago de Cali, Santiago de Cali 760001, Colombia; 4 Facultad de Medicina, Universidad Militar Nueva Granada, Bogotá 1101111, Colombia; 5 Clínica de Marly, Bogotá 110231, Colombia; 6 Masira Research Institute, Universidad de Santander (UDES), Bucaramanga 680006, Colombia; 7 Population Health Research Institute, McMaster University, Hamilton, ON L8S 4K1, Canada

**Keywords:** COVID-19, SARS-CoV-2, respiratory function tests, outpatient, hospitalized, sequelae

## Abstract

**Background**: The factors driving Coronavirus disease 2019 (COVID-19) severity and its long-term respiratory sequelae remain poorly understood. This study evaluates whether baseline lung function (LF) influences COVID-related clinical outcomes, mortality, and post-infection LF decline. **Methods**: Data from 602 participants in the Prospective Urban Rural Epidemiology (PURE)-Colombia study were analyzed. Among these, 200 with confirmed SARS-CoV-2 infection and 402 controls (65% women; 68% aged ≥60 years). All underwent baseline spirometry prior to 2010 and follow-up testing 1–40 months post-recovery. Among infected individuals, 51 (26%) died. Spirometric parameters Forced Expiratory Volume in 1 Second (FEV_1_), Forced Vital Capacity (FVC), and Peak Expiratory Flow (PEF) were compared using paired *t*-tests and Cohen’s d. Non-parametric data were compared using Wilcoxon s (z statistic). **Results**: Compared to baseline LF, hospitalized COVID-19 patients showed significant declines in follow-up LF: FEV_1_ (2.84 vs. 2.34 L; *p* = 0.002), FVC (3.01 vs. 2.53 L; *p* = 0.006), and PEF (399 vs. 328 L; *p* = 0.001). Non-hospitalized COVID-19 cases showed a non-significant downward trend, while controls maintained stable LF. Risk factors for post-COVID FEV_1_ < 80% predicted included hospitalization, elevated waist-to-hip ratio, and incomplete or absent COVID-19 vaccination. Moderate-to-high physical activity was protective. Post-COVID PEF < 80% predicted was associated with female sex, diabetes mellitus, and subsidized healthcare enrollment. Mortality risk was elevated among individuals with low baseline LF, age > 65, male sex, hypertension, obesity, low physical activity, and reduced handgrip strength. **Conclusions**: Significant LF decline was observed in hospitalized COVID-19 patients, with minimal changes in outpatients and controls. Identifying clinical and demographic predictors of post-COVID LF impairment may inform targeted interventions to mitigate long-term pulmonary complications.

## 1. Introduction

Coronavirus disease 2019 (COVID-19) primarily targets the lungs, triggering a wide range of acute and chronic respiratory complications, from transient dysfunction to chronic decline [1]. Although persistent pulmonary abnormalities following hospitalization have been well-documented, often lasting months or years, the determinants of clinical severity and long-term respiratory outcomes remain poorly defined [2,3,4,5]. Most existing studies enrolled participants only after SARS-CoV-2, lacking baseline clinical data such as pre-infection lung function (LF) [6,7,8,9,10,11,12,13]. As a result, the extent of infection-related LF decline and the premorbid factors associated with adverse pulmonary sequalae post COVID-19 remain insufficiently characterized.

COVID-19 severity is strongly linked to LF abnormalities, particularly restrictive patterns predominantly observed in older adults and individuals with comorbid conditions [6,14]. These impairments are frequently linked to post-infection pulmonary fibrosis, characterized by reduced Diffusing Capacity (DLCO) and Total Lung Capacity (TLC) [7,8,9,15,16]. *Post-mortem* analysis of severe COVID-19 pneumonia has consistently revealed extensive fibrotic damage [15,17,18]. Moreover, pre-existing LF impairment due to chronic lung disease increases the risk of respiratory failure and mortality. In such individuals, COVID-19 tends to present more severely, and baseline LF deficits may significantly confound the post COVID-19 LF findings [19].

The primary aim of this study was to assess changes in LF following varying severities of symptomatic SARS-CoV-2 infections in a well-characterized, community-based Colombian cohort enrolled before the COVID-19 pandemic. The availability of comprehensive baseline data, including pre-infection spirometry, provides a unique opportunity to estimate LF decline attributable to SARS-CoV-2 infection. A secondary objective was to identify baseline predictors of adverse outcomes, including persistent LF impairment following SARS-CoV-2 infection. We hypothesized that symptomatic SARS-CoV-2 infection would be associated with a decline in pulmonary function, particularly among individuals with more severe disease, and that baseline characteristics would serve as predictors of persistent impairment.

The Prospective Urban Rural Epidemiology (PURE)-Colombia cohort included 7552 adults aged 30–70 years, recruited between 2003 and 2009, with ongoing follow-up. Designed to reflect geographical and social diversity of the country, the PURE cohort includes participants from urban and rural areas across 11 of Colombia’s most populous departments (Atlántico, Bolívar, Caldas, Casanare, Cauca, Cesar, Cundinamarca, Nariño, Quindío, Santander, and Tolima), home to over a half the national population [20]. Its large sample size and broad representation reduce the selection bias and enable identification of universal risk factors, overcoming limitations of more homogeneous populations.

## 2. Materials and Methods

### 2.1. Sample

**Figure 1:**
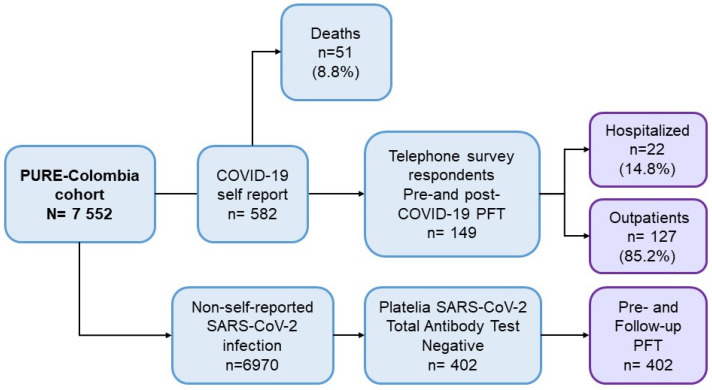
Flowchart of PURE-Colombia cohort participants with pre- and post-COVID-19 LF tests.

This study is part of the PURE-Colombia prospective cohort study. From an initial PURE-Colombia cohort of 7552 individuals, 582 participants were selected based on confirmed SARS-CoV-2 infection by RT-PCR or antigen (Ag) testing in symptomatic individuals between March 2020 and June 2022. Of these subjects, 51 died from this cause (8.8%), and 149 had available baseline pulmonary function test (PFT) data from before 2020 and post-COVID-19. Each participant underwent two spirometry assessments: baseline measurements between 2006 and 2009 (pre-COVID-19) and follow-up measurements between 2021 and 2022 (post-COVID-19). FEV_1_, FVC, FEV_1_/FVC ratio, and PEF were assessed. Of the 149 survivors, 127 (85.2%) received outpatient treatment, while 22 (14.8%) required hospitalization in general wards, intermediate care units, or intensive care units (ICUs) (Figure 1).

Additionally, 402 non-infected PURE cohort participants were included as a control group to compare baseline characteristics and LF changes between infected and non-infected groups. Control individuals were selected based on a negative Platelia SARS-CoV-2 Total Ab test (Bio-Rad, Marnes-la-Coquette, France) and available LF data. The follow-up spirometry was performed at the same time as the blood sample was collected for the SARS-CoV-2 antibody test.

### 2.2. Data Collection

Baseline data were collected from all participants between January 2005 and December 2009. Follow-up data were collected from all surviving participants and the control group between January 2021 and May 2022. Consequently, in the infected group, follow-up measurements were conducted between 1 and 40 months after SARS-CoV-2 infection. Data collected during visits included standardized questionnaires to obtain information on sociodemographic, vaccination (complete, incomplete, unvaccinated), smoking status, physical activity level (<150, 150–750, >750 min/week) [21,22], and comorbidities. In addition, laboratory tests (creatinine, cholesterol, non-HDL cholesterol, triglycerides) and clinical assessments including repeat spirometry, body mass index (BMI), waist-to-hip ratio (WHR), blood pressure, and handgrip strength (HS) were also recorded.

### 2.3. Spirometry

LF measurements were conducted using the MicroGP (MicroMedical, Chatham, IL, USA) portable spirometer at baseline, and the EasyOne Plus (Ndd, Medical Technologies, Zurich, Switzerland) during follow-up, using a standardized protocol and international guideline recommendations from the American Thoracic Society (ATS) and the European Respiratory Society (ERS) [23]. Sex, race, date of birth, height, weight, tobacco consumption description and history of asthma were recorded prior to test. Participants were trained to perform up to eight maximal pre-bronchodilator forced expiratory maneuvers while standing and wearing a nose clip. Maneuvers were supervised by a trained operator to ensure maximal effort, ≥6 s of exhalation, absence of coughing and, if possible, the achievement of a minimum of three acceptable FEV1 and FVC or one in the individuals unable to perform multiple attempts. Participants with at least 1 acceptable maximal effort were included. The highest FEV_1_, FVC, and PEF were analyzed. Spirometry data were centrally checked and validated for quality [24,25].

Lung function predicted values were calculated using the neutral Global Lung Initiative (GLI) equations based on sex at birth, age, and height [26]. Spirometric impairment was categorized using the following cut-offs for predicted values: FEV_1_ < 80%, FVC < 80%, FEV_1_/FVC ratio < 70%, and PEF < 80%.

### 2.4. Handgrip Strength (HS)

HS was assessed using a Jamar dynamometer (Sammons Preston, Bolingbrook, IL, USA). Participants performed three maximal squeezes per hand—each lasting 3 s. Measurements were taken with elbow flexed at 90° and arm at the side. The device was adjusted for hand size, and HS was recorded in kilogram-force (kgf). The highest measurement from the dominant hand was selected for analysis. Reference values were drawn from a healthy sub-population within the PURE cohort study by Leong et al. [27], according to age, sex at birth, and geographic region for South American populations.

### 2.5. Data Analysis

Descriptive statistics were used to characterize the study population. Categorical variables were summarized as frequencies and percentages, and continuous variables as means and standard deviations or medians and interquartile ranges. Group comparisons were conducted using Chi^2^ tests for categorical variables and Student’s *t*-tests form normally distributed continuous variables with a significant level of 0.05 and 95% confidence intervals (CI).

Pre- and post-COVID-19 LF were compared using paired Student’s *t*-tests for normally distributed variables, and effect sizes reported as Cohen’s d. For non-normal distributed variables, Wilcoxon test (z statistic) was applied, and effect sizes expressed as paired rank biserial correlations. Effect magnitude was interpreted as small (0.20), medium (0.50), and large (0.80) [28]. McNemar’s Test was used to assess changes in spirometric parameter proportions.

Although generalized linear mixed models (GLMMs) are suitable for analyzing longitudinal data with multiple repeated measurements per subject, their application in this study was not appropriate due to the nature of the data and the variables of interest. First, the design included only two measurements (pre- and post-COVID-19), which resulted in a minimal intra-subject correlation structure. In addition, spirometric parameters did not meet the assumption of normality, even after applying several transformations, thereby compromising the validity of a linear model with normally distributed errors.

Logistic regression was used to assess the association of sociodemographic and clinical factors with two post-COVID-19 LF outcomes: FEV_1_ < 80% predicted and PEF < 80% predicted. Consequently, only participants with normal baseline spirometry (>80% predicted) were included. Variables with *p*-value ≤ 0.25 in univariate analysis were entered into multivariate models using stepwise backward elimination (entry *p* < 0.10; removal *p* > 0.25). Model fit was evaluated using Hosmer–Lemeshow test and likelihood ratio tests. Results were reported as odds ratios (OR) with 95% CI and *p*-values.

Multiple linear regression was used to identify predictors of LF parameters (such as FEV_1_, FVC, FEV_1_/FVC ratio, and PEF) treating each as a dependent variable in separate models. Independent variables were selected based on prior evidence and biological plausibility. Assumptions of linearity, homoscedasticity, independence, and normality residuals were verified. Multicollinearity was assessed via variance inflation factor (VIF); variables with high VIF were excluded or transformed. Regression outputs included β coefficients, standard errors, 95% CI, and *p*-values.

Spearman’s correlation was used to explore association between COVID-19 severity (outpatient, hospitalized, deceased) and variables such as sex at birth, age, BMI, WHR, physical activity, vaccination status, and comorbidities. Correlation strength was classified as: very low (<0.20), low (0.20–0.39), moderate (0.40–0.69), high (0.70–0.90), and very high (>0.90). All analyses were performed using STATA 17.0^®^ statistical software (StataCorp, College Station, TX, USA).

### 2.6. Ethical Aspects

The PURE-Colombia project received approval from local ethics committees, and participants provided informed consent by signing the necessary documents. Additionally, the nested project “Prediction Models of COVID-19 Severity Using Grip Strength, Biomarkers, and Clinical-Epidemiological Background in the PURE-Colombia Cohort” was approved by the Institutional Bioethics Committee of the Universidad de Santander (UDES) (Act N° 06/8 March 2022).

We used the STROBE reporting guideline [29] to draft this manuscript, and the STROBE reporting checklist [29] when editing (see Supplementary Materials Table S1).

## 3. Results

### 3.1. Demographic Characteristics

There were 551 participants with acceptable spirometry and complete baseline data included in the analysis. The baseline characteristics of the control group and those with SARS-CoV-2 infection according to severity are provided in Table 1. There were more females (65.7%), participants aged 60 years or older (68.4%), and low educational attainment (67.7%) in the overall cohort. Over half resided in urban areas (57%) and were covered by subsidized health insurance (51%). Only 22% had completed a SARS-CoV-2 vaccination schedule (one Janssen dose or two doses of other vaccines). The majority were overweight or obese (59%), 40% had elevated WHR, 35% were smokers, and 32% had hypertension. Nearly half (50%) were engaged in high levels of physical activity (>750 min/week) and 69% had normal HS. Mean levels of creatinine, cholesterol, and triglyceride were within normal ranges.

Vaccination coverage was similar between hospitalized (21%) and outpatient (23%) survivors but notably lower among deceased individuals (14%). Compared to survivors (*n* = 149), deceased participants (*n* = 51) were more likely to be males, aged ≥60 years, smokers, hypertensive, with non-O blood types, reduced HS, received incomplete or no COVID-19 vaccination, and had elevated serum creatinine. These differences between COVID-19 deceased and survivors were statistically significant (see Supplementary Materials Table S2). Unvaccinated status was more prevalent among hospitalized (68.5%) and deceased (72.6%) individuals. The most administered vaccines were Pfizer, AstraZeneca, and Sinovac (see Supplementary Materials Tables S3 and S4).

### 3.2. Post-COVID-19 Spirometry

Spirometry measurements were compared across the three groups: individuals with and without SARS-CoV-2 infection, and within the infected group, between hospitalized and outpatient cases. Among COVID-19 survivors (*n* = 149), 38.9% (*n* = 58) had follow-up spirometry performed within 12 months, and 61.2% (*n* = 91) had spirometry performed after 12 months of infection. The median time to post-infection spirometry assessment was 13 months [interquartile range (IQR) 10–18]. The distribution in the timing of spirometry assessments was similar between the outpatient and inpatient groups, with most assessments occurring after the first year. In this regard, the data were organized into five categories, each representing a six-month period. The largest proportion of assessment occurred between 13 and 18 months (37.8% for outpatient vs. 40.9% for hospitalized), followed by 7–12 months (22.8% and 22.7%, respectively). Smaller proportions were observed in the 1–6 month and >24-month categories, with no divergent patterns. Statistical comparison using the chi-square test confirmed no significant differences between groups across time categories (*p* = 0.99). Similarly, when dichotomized at 12 months, 60.6% of outpatients and 63.6% of hospitalized patients underwent PFTs after 12 months, again without significant differences (*p* = 0.98).

**Figure 2:**
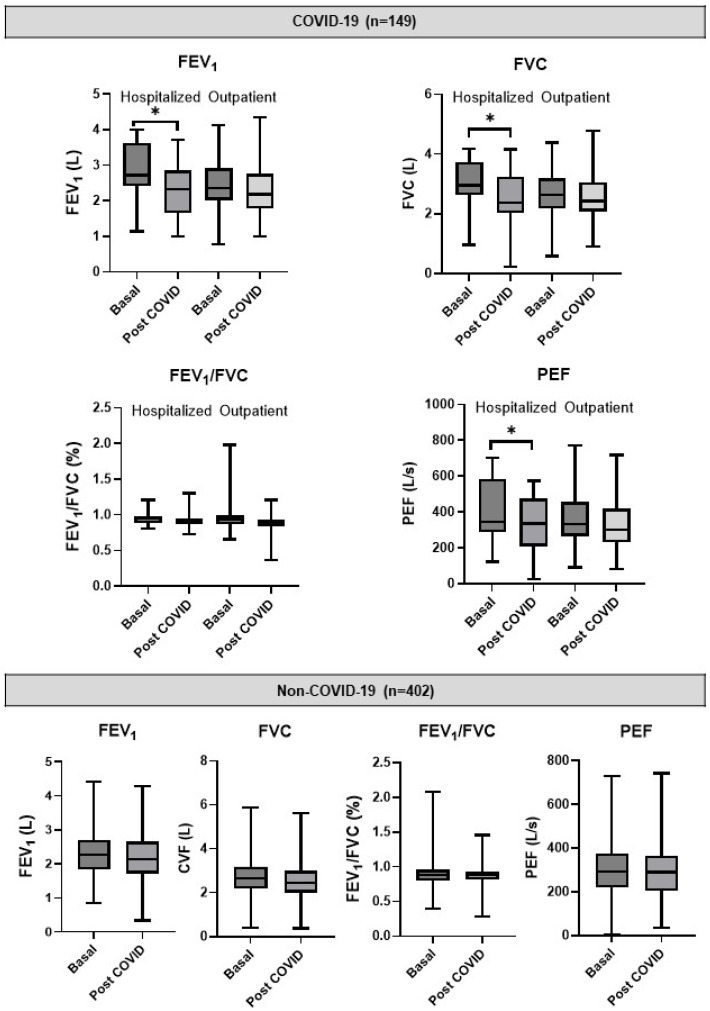
Pre- and post-lung function parameters in COVID-19 and control participants. FEV_1_: Forced Expiratory Volume in 1 Second; FVC: Forced Vital Capacity; PEF: Peak Expiratory Flow (PEF); PFT: Pulmonary Function Test. * Significant differences: paired Student’s *t*-test, *p* < 0.05.

The outpatient group showed no significant change between baseline and post-infection spirometry values in PEF (363 vs. 354 L/s, *p* = 0.109), FEV_1_ (2.43 vs. 2.41 L, *p* = 0.23), and FVC (2.61 L vs. 2.60, *p* = 0.43). In contrast, hospitalized cases exhibited significant decline in post infection values compared to baseline in PEF (399 vs. 329 L/s, *p* = 0.001), FEV_1_ (2.84 vs. 2.34 L, *p* = 0.002), and FVC (3.01 vs. 2.53 L, *p* = 0.006). It is striking that the average baseline LF parameters were higher in the hospitalized group compared to the outpatient group, possibly related to the higher proportion of men in the hospitalized group (50.0% vs. 33.9%). Effect size analysis showed no impact on the FEV_1_/FVC ratio, a small effect on the PEF, and a moderate effect on the FEV_1_ and FVC. No significant change was observed between baseline and follow-up spirometry measurements in the control group (Figure 2). Detailed information on measures of central tendency and dispersion of LFT can be found in the Supplementary Materials (Table S5).

### 3.3. Predicted Values of LFT

**Figure 3:**
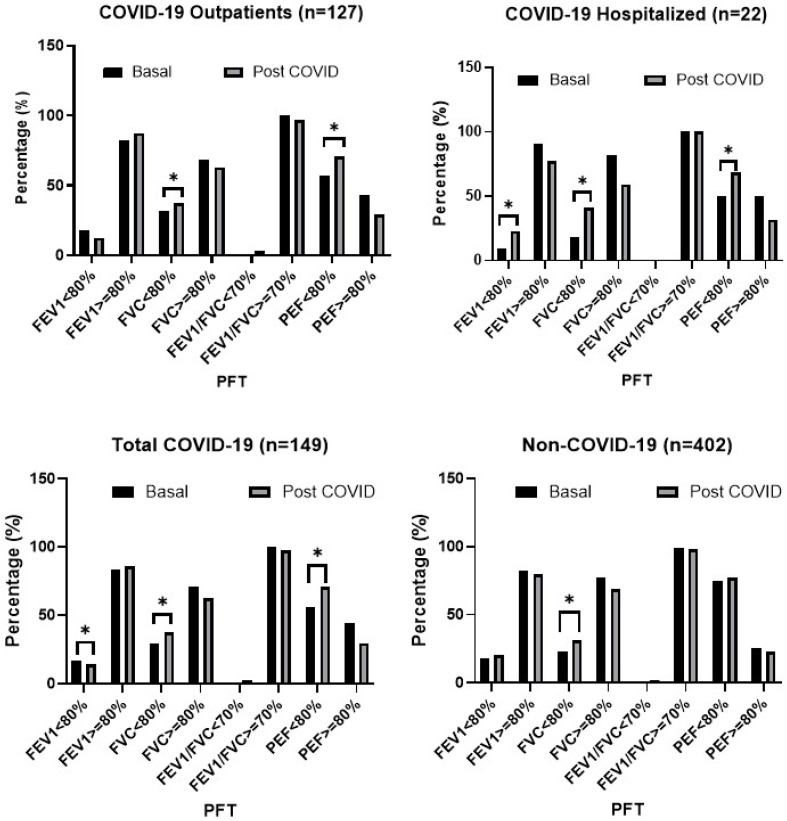
Values of pre- and post-COVID lung function (LF) in outpatient and hospitalized participants. Data expressed are the proportion of patients in each category of LF stratified according to <80% predicted versus ≥80% predicted. FEV_1_: Forced Expiratory Volume in 1 Second; FVC: Forced Vital Capacity; PEF: Peak Expiratory Flow (PEF); PFT: Pulmonary Function Test. * Significant differences (*p* < 0.05).

When compared with age-, sex-, and height-adjusted reference values, the proportion of spirometry impairment, using a cut-off of 80% of predicted, showed a significant increase among individuals who had COVID-19. In the outpatient group, there was an increase in the proportion of FVC and PEF impairment after COVID-19. In the hospitalized group, the proportion of FEV_1_, FVC and PEF impairment increased after COVID-19 (Figure 3), while the FEV_1_/FVC ratio remained unchanged.

The percentages of spirometry impairment also differ according to the timing of spirometry assessment following infection. For example, 75% of outpatient cases had PEF < 80% predicted within 12 months of infection, which decreased to 10% beyond the first year. In contrast, 75% of hospitalized cases showed PEF < 80% predicted beyond one year of infection, while 57.1% had FVC < 80% predicted within the first year. In non-infected individuals, only FVC showed a significant change between baseline and follow-up.

**Figure 4:**
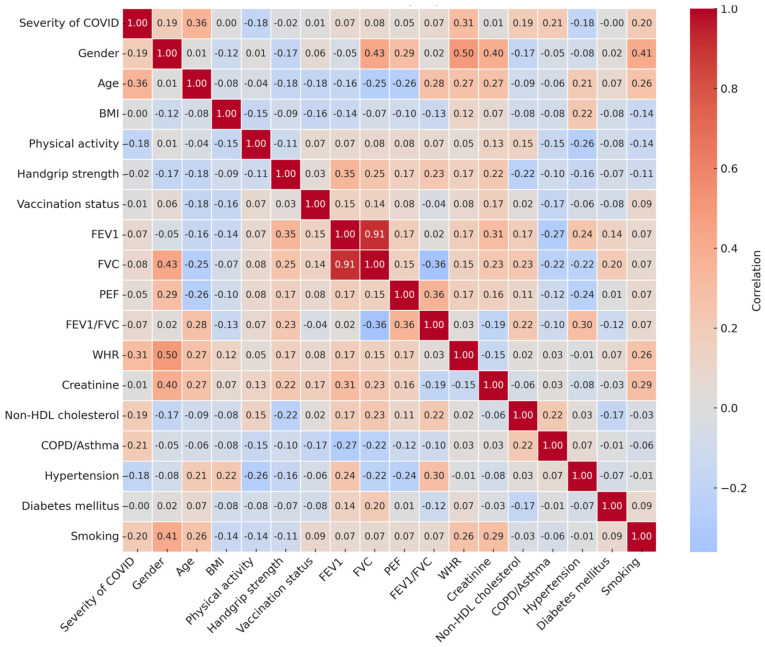
Correlation matrix with COVID severity. COVID-19 severity was classified into three categories: outpatient, hospitalized, and deceased. BMI: Body Mass Index; FEV_1_: Forced Expiratory Volume in 1 Second; FVC: Forced Vital Capacity; PEF: Peak Expiratory Flow (PEF); PFT: Pulmonary Function Test; WHR: Waist-to-Hip Ratio; COPD: Chronic Obstructive Pulmonary Disease.

Further analysis showed that individuals who died had significantly lower pre–COVID FVC and FEV_1_ values (see Supplementary Materials Tables S6 and S7). Correlation analysis revealed low-to-moderate associations between COVID-19 severity with age, WHR, COPD/asthma history, and smoking. Spirometry parameters also correlated with HS and sex (Figure 4).

### 3.4. Association Between Sociodemographic and Clinical Characteristics with Post-COVID LF

Associations between baseline characteristics with post-COVID LF decline were analyzed in two subgroups: individuals with baseline FEV_1_ > 80% (*n* = 128); and those with a baseline PEF > 80% (*n* = 66) (Table 2). The likelihood of post-COVID FEV_1_ impairment (<80% predicted) was higher among hospitalized participants, and in those with low physical activity, elevated WHR, and incomplete or no COVID-19 vaccination. Similar analysis revealed that the risk of post COVID PEF impairment (<80% predicted) was higher among females, those with diabetes mellitus, and participants in the subsidized healthcare system. No significant association was found with post-COVID FVC < 80% predicted. All models were adjusted for age, sex, BMI, WHR, COPD/asthma history, smoking, and timing of LFT.

Multiple linear regression was initially considered to identify predictors of LF parameters. However, residuals failed to meet normality assumptions despite variables transformations and adjustments, as confirmed by Q–Q plots, residual scatter plots, and the Shapiro–Wilk test. To avoid biased estimates, multivariate regression was not pursued. Instead, descriptive and bivariate analyses were used to explore associations between key variables.

### 3.5. Association Between Sociodemographic and Clinical Characteristics with Mortality

The multivariable model for mortality demonstrated strong performance (Hosmer–Lemeshow Chi^2^ = 147.83; *p* = 0.973; Pseudo R^2^ = 0.407; AUC = 0.902). Significant predictors of COVID-19 mortality included male sex, age > 65 years, hypertension, obesity (BMI > 30 kg/m^2^), low physical activity, decreased HS, and baseline FEV_1_ < 80% predicted. These findings emphasize the predictive value of age, sex, comorbidities, and physical fitness in mortality risk, and highlight the importance of preserved LF and muscle strength in avoiding poor health outcomes (Table 3).

## 4. Discussion

Although post-COVID-19 lung dysfunction is recognized, few studies have used pre-infection spirometry for comparison [11,30,31], leaving the true impact of acute SARS-CoV-2 infection on LF poorly defined. This study included PURE-Colombia cohort participants with spirometry data both before and after SARS-CoV-2 infection. Changes in LF parameters (FEV_1_, FVC, FEV_1_/FVC ratio, and PEF) were assessed within individuals over time and compared across three groups: (1) SARS-CoV-2-naïve individuals, (2) confirmed cases managed as outpatients, and (3) cases requiring hospitalization, including general wards, intermediate care, or ICU.

This study provides a unique opportunity to evaluate longitudinal changes in LF within a well-characterized, population-based cohort which was systematically recruited prior to the COVID-19 pandemic. The PURE-Colombia cohort is a well characterized sample of the general population (anthropometry, HS, comorbidities, physical activity, and biochemical markers), which allows for robust analysis of baseline factors associated with post-infection LF decline. By incorporating both symptomatic and asymptomatic individuals with confirmed SARS-CoV-2 infection, the study reduces the risk of detection bias. It further strengthens its design by comparing spirometric parameters between infected participants and uninfected controls. According to the available literature this is the first study to report SARS-CoV-2-associated pulmonary alterations in a Colombian population.

In our study, hospitalized patients with moderate to severe COVID-19 exhibited significant reductions in FEV_1_ (~500 mL), FVC (~480 mL), and PEF (~70 L), indicating persistent pulmonary involvement, even beyond one year following the index infection. In contrast, outpatient cases showed minor, non-significant decline in spirometry, primarily in PEF. As controls, we observed no significant changes between baseline and follow-up spirometry in non-infected individuals during the same period (Table 2). These results are consistent with prior studies which show that even non-hospitalized individuals or subclinical infections demonstrate measurable decline in lung volume post-COVID, particularly in FVC, even though prior vaccination against SARS-CoV-2 offered some protection [30,32]. This aligns with our findings, which showed vaccinated individuals were less likely to require hospitalization following SARS-CoV-2 infection [33].

Similarly, the BAMSE Sweden cohort study conducted in 853 young adults (mean age 22–24), reported no significant differences in LF measurements (FEV_1_, FVC, or FEV_1_/FVC) before and during the pandemic in 243 seropositive participants (29%) compared to seronegative peers. Note that, in this cohort, none of the seropositive individuals were hospitalized, suggesting mild or asymptomatic infections. Pre-existing asthma, allergic sensitization, or corticosteroid use had no measurable impact on LF [24]. Other studies also reported acute decline in FEV_1_ (~100 mL) and FVC (~150 mL) three months post-COVID in non-hospitalized individuals [34,35].

In contrast, Mo et al. [36] evaluated 110 hospitalized COVID-19 patients at discharge, comparing them to healthy controls. They reported that 47.2% of the cases had reduced DLCO (<80% predicted), 25.5% had decreased FVC, and 13.7% had reduced FEV_1_—findings more common in moderate to severe cases. Also, a systematic review of RT-PCR-confirmed cases without control group found that approximately 40% of individuals had reduced DLCO, 15% showed restrictive patterns, and 7% had obstructive changes [14]. These results highlight substantial residual pulmonary impairment in nearly half of hospitalized patients, reinforcing the need for long-term respiratory follow-up and rehabilitation.

Our study identified several factors associated with post-COVID-19 declines in FEV_1_, and FVC. These include hospitalization during the acute illness, female sex, diabetes mellitus, elevated WHR, low physical activity, incomplete or absent COVID-19 vaccination, and affiliation with the subsidized health system. These results align with prior studies by Shah et al. [37] which also reported that women may experience more severe pulmonary complications from COVID-19, potentially due to differences in immune response, hormonal factors, anatomy, and underlying conditions.

Unlike previous studies that evaluated LF at fixed post-infection intervals, this study followed a flexible assessments schedule determined by the cohort’s regular follow-up visits rather than by the timing of SARS-CoV-2 infection. This approach aligned with individual recovery trajectories but limited direct comparison with previous research. However, when stratified by timing, greater PEF impairment was observed within the first 12 months among outpatients, while hospitalized individuals showed more pronounced PEF decline after one year. FVC impairment was also more frequent in hospitalized patients during the first-year post-infection (57.1%) compared to outpatients (36.4%). Notably, FVC values one year after COVID-19 in hospitalized patients, were significantly lower than those observed post-COVID outpatients (57.1% vs. 12.5%).

Similarly, in another prospective study of 301 individuals with confirmed COVID-19, both hospitalized and non-hospitalized participants were assessed using spirometry and DLCO over a 12-month follow-up. At one year, 25% showed persistent impairment, which increased by disease severity: 11% in mild cases, 22% in moderate, and 48% in severe or critical cases. DLCO values improved progressively over time [38]. Likewise, Rosas et al. [39] in the LOPAC study, found that 32.4% of 173 hospitalized patients had persistent pulmonary abnormalities at 12 months, despite stable FVC and FEV_1_. An inverse correlation between DLCO and CT-detected structural changes suggested persistent and long-lasting lung damage [39].

Our results showed that delayed recovery of LF was significantly associated with older age, ≥3 comorbidities (*p* < 0.001), and severe acute presentation (*p* < 0.001), even after adjusting for age and sex. These findings underscore the long-term impact of moderate to severe COVID-19 and the need for targeted rehabilitation strategies [30]. In this regard, Chai et al. [40] and Larsson et al. [41] linked persistent LF abnormalities, after one year, to female sex, fatigue, older age, cardiovascular disease, and radiographic findings such as ground-glass opacities. Similarly, in a two-year follow-up of 172 patients’ post-hospitalization, 14% had ongoing ventilatory or diffusion defects, with age, male sex, and obesity as key risk factors [42]. During the three years, although radiological and functional improvements were noted, over one-third continued to exhibit respiratory symptoms and reduced diffusion capacity [37].

Given the protective role of moderate-to-high physical activity against LF decline, our findings highlight the importance of implementing physical rehabilitation programs for individuals recovering from COVID-19, especially those with comorbidities or who required hospitalization. Accordingly, previous studies described that after 12 months of follow-up, significant improvements in FEV_1_, FVC, and PEF were observed alongside increased diaphragm thickness and mobility during maximal inspiration [43,44]. Gains in muscle strength were also reflected in enhanced HS and inspiratory/expiratory pressures [45]. However, persistent symptoms such as musculoskeletal pain, fatigue, and dyspnea, may remain despite spirometric recovery, highlighting the need for ongoing clinical and radiological monitoring, especially in moderate to severe cases [7,46]. Supporting our findings on physical activity, a study by Amaral et al. [47] linked dynapenia in Long COVID patients to impaired LF, reduced respiratory muscle strength, and diminished exercise capacity. Decreased inspiratory muscle strength has also been associated with persistent dyspnea [48]. These insights reinforced the importance of rehabilitation programs focused on improving aerobic capacity, LF, and respiratory muscle strength in post-COVID recovery [49].

In this study, comorbidities were associated with LF deterioration and delayed recovery. Previously, comorbidities such as diabetes, hypertension, cardiovascular disease, obesity, chronic kidney disease, and chronic pulmonary conditions were consistently linked to higher COVID-19 mortality, particularly in older adults and those with multiple conditions. Combined with evidence supporting the protective effect of vaccination, our findings reinforce a multifactorial model of COVID-19 mortality, shaped by underlying health status and vaccine coverage [50,51]. Our findings indicate that individuals over 65 years of age, males, and those with hypertension, obesity, or low physical activity levels have a significantly higher risk of mortality. Conversely, preserved lung function and adequate muscle strength emerged as protective factors associated with lower mortality risk.

Lower HS has been consistently linked to higher mortality and severe outcomes in COVID-19 patients, including increased risk of intubation, prolonged hospitalization, and death. In large cohorts like the UK Biobank higher HS, male sex and obesity was associated with reduced risk of severe COVID-19. These findings highlight muscle strength, as a marker of functional reserve and overall health as a valuable prognostic indicator, independent of muscle mass [52,53,54,55].

Future research should validate these findings in larger and more diverse cohorts, while exploring the long-term trajectories of pulmonary function recovery. Longitudinal studies incorporating extended follow-up, stratification by disease severity, and integration of baseline clinical and demographic factors will be essential for identifying predictors of sustained impairment. Such investigations could consolidate the role of PFT as a cost-effective and accessible tool to guide post-COVID monitoring strategies. Establishing routine follow-up would strengthen risk stratification and enable timely, targeted interventions to mitigate long-term respiratory sequelae. Moreover, accurately estimating the true burden of COVID-19 on respiratory health is fundamental to designing comprehensive rehabilitation programs. These should integrate multidisciplinary strategies such as respiratory training, physical reconditioning, nutritional support, and metabolic control to optimize long-term outcomes. From a public health perspective, evaluating clinical and socioeconomic determinants, together with the cost-effectiveness of structured follow-up and early intervention programs, will be critical to inform clinical practice and health policy, particularly in low- and middle-income settings where resources are limited.

Limitations of this study include incomplete spirometry coverage among COVID-19 cases and variable timing of post-COVID-19 assessments. Nevertheless, the interval between COVID-19 diagnosis and spirometry testing was precisely recorded. It was also not possible to determine whether the acute SARS-CoV-2 infection was a first or recurrent episode. Additionally, limited access to healthcare, especially ICU care during the pandemic and absence of medical records restricted accurate classification of COVID-19 severity.

## 5. Conclusions

This cohort study demonstrated that adults hospitalized for SARS-CoV-2 infection experienced significant decline in FEV_1_ (~500 mL), FVC (~480 mL), and PEF (~70 L) when comparing pre- and post-COVID LF. No significant changes were observed in non-infected controls or in outpatients, though the latter showed a non-significant decline. Key risk factors for post-COVID-19 FEV_1_ < 80% predicted included hospitalization, elevated WHR, low physical activity, and incomplete or absent SARS-CoV-2 vaccination. For PEF < 80% predictive, increased risk was associated with female sex, diabetes mellitus, and enrollment in the subsidized healthcare system. Identifying these predictors of post-COVID LF decline can help prioritize individuals for targeted follow-up and early intervention. Mortality was significantly linked to older age, male sex, hypertension, obesity, and low physical activity. Conversely, preserved LF and muscle strength reduced risk, underscoring the predictive value of both clinical and functional indicators in assessing mortality.

## Figures and Tables

**Table 1:** Demographic and clinical baseline data of the study population.

Variable		Non-COVID-19 (*n* = 402)		COVID-19 (*n* = 149)						Total Population (*n* = 551)		*p*-Value *
				Outpatients (*n* = 127)		Hospitalized (*n* = 22)		Total (*n* = 149)				
		*n*	%	*n*	%	*n*	%	*n*	%	*n*	%	
Gender	Female	267	66.4	84	66.1	11	50.0	95	63.8	362	65.7	0.629
	Male	135	33.6	43	33.9	11	50.0	54	36.2	189	34.3	
Age	<60 years	109	27.1	58	45.7	7	31.8	65	43.6	174	31.6	**<0.001**
	≥60 years	293	72.9	69	54.3	15	68.2	84	56.4	377	68.4	
Educational level	Low ^1^	295	73.4	64	50.4	14	63.6	78	52.3	373	67.7	**<0.001**
	Middle ^2^	71	17.7	30	23.6	4	18.2	34	22.8	105	19.1	
	High ^3^	36	8.9	33	26.0	4	18.2	37	24.8	73	13.2	
Health insurance ^4^	Contributory	182	45.3	80	63.0	8	36.4	88	59.1	270	49.0	**0.005**
	Subsidized ^4^	220	54.7	47	37.0	14	63.6	61	40.9	281	51.0	
Place of residence	Urban	246	61.2	57	44.9	13	59.1	70	47.0	316	57.4	**0.003**
	Rural	156	38.8	70	55.1	9	40.9	79	53.0	235	42.6	
Body mass index (BMI) (kg/m^2^)	Normal: 18.5–24.9	174	43.3	47	37.0	4	18.2	51	34.2	225	40.8	0.068
	Overweight: 25–29.9	165	41.0	56	44.1	13	59.1	69	46.3	234	42.5	
	Obesity: ≥30	63	15.7	24	18.9	5	22.7	29	19.5	92	16.7	
Background	Smoking	139	34.6	46	36.2	7	31.8	53	35.6	192	34.8	0.907
	COPD/asthma	20	5.0	2	1.6	4	18.2	6	4.0	26	4.7	0.810
	Hypertension	136	33.8	35	27.6	8	36.4	43	28.9	179	32.5	0.315
	Diabetes mellitus	81	20.1	13	10.2	2	9.1	15	10.1	96	17.4	**0.008**
Physical activity level (min/week)	Mild < 150	49	12.2	16	12.6	8	36.4	24	16.1	73	13.2	0.287
	Moderate 150–750	146	36.3	50	39.4	8	36.4	58	38.9	204	37.0	
	High > 750	207	51.5	61	48.0	6	27.3	67	45.0	274	49.7	
Waist-to-hip ratio (WHR)	Normal ^5^	250	62.2	72	56.7	6	27.3	78	52.3	328	59.5	**0.046**
	High	152	37.8	55	43.3	16	72.7	71	47.7	123	40.5	
Handgrip strength (kg)	Normal	270	67.2	95	74.8	15	68.2	110	73.8	380	69.0	0.162
	Decreased ^6^	132	32.8	32	25.2	7	31.8	39	26.2	171	31.0	
Vaccination status prior to COVID-19	Complete scheme ^7^	93	23.1	116	91.3	14	63.6	31	20.8	124	22.5	0.640
	No vaccine dose	289	71.9	6	4.7	6	27.3	100	67.1	389	70.6	
	Incomplete scheme	20	5.0	5	3.9	2	9.1	18	12.1	38	6.9	
Laboratories (mg/dL) (mean ± SD)	Creatinine	0.9 ± 0.2		0.99 ± 0.24		0.97 ± 0.22		0.9 ± 0.2		0.9 ± 0.2		**<0.001**
	Non-HDL cholesterol	161.8 ± 45.7		152.0 ± 44.0		170.0 ± 33.0		154.0 ± 43.1		160.5 ± 45.2		0.079
	Triglycerides	174.1 ± 86.8		168.0 ± 76.0		175 ± 120		169.0 ± 84.0		173.2 ± 86.1		0.527

^1^ Low: no education, primary education, lower secondary education; ^2^ Middle: complete secondary education; ^3^ High: technician, bachelor, master, doctoral or equivalent; ^4^ Colombia’s compulsory health insurance regime. ^5^ Women: 0.71–0.85 cm; Men: 0.78–0.94. ^6^ Women: <19 kg; Men: <32 kg. ^7^ One dose of Janssen vaccine and two doses of the other vaccines. * Differences between the group of subjects without COVID-19 and the total group of subjects with COVID-19. The bold formatting in solme values from the “*p*-Value *” indicates “statis-tically significant value”.

**Table 2:** Factors associated with reduced post-COVID-19 predicted values in LF tests.

Variable	Crude OR	Adjusted OR	CI_95%_	*p*-Value
FEV_1_ (L) < 80% predicted *				
Hospital management during COVID-19	3.82	4.97	1.95–38.63	0.047
Low level of physical activity	3.54	3.85	1.10–12.50	0.039
High waist-to-hip ratio	2.12	1.65	1.53–22.34	0.040
No prior or incomplete vaccination against COVID-19	5.03	4.21	1.88–18.75	0.044
Pseudo R^2^ = 0.4026. Hosmer–Lemeshow Chi^2^ 170.57 (*p* ≤ 0.001). AUC = 0.892				
PEF (L/s) < 80% predicted *				
Diabetes mellitus	8.96	7.55	1.93–61.02	0.048
Female gender	7.84	6.91	1.44–33.01	0.015
Health insurance: Subsidized	3.78	3.53	1.12–11.16	0.031
Pseudo R^2^ = 0.2646. Hosmer–Lemeshow Chi^2^ 111.00 (*p* ≤ 0.001). AUC = 0.848				

OR: odds ratio; CI: confidence interval; PEF: Peak Expiratory Flow; FEV_1_: Forced Expiratory Volume in 1 s; * Models adjusted for age, gender, body mass index, waist-to-hip ratio, history of COPD/asthma, smoking, and completion time of the pulmonary function tests (≤12 months vs. >12 months).

**Table 3:** Baseline characteristics associated with COVID-mortality.

Variable	Crude OR	Adjusted OR	CI_95%_	*p*-Value
Male sex	4.87	5.99	1.81–19.75	0.003
Age > 65 years	10.45	8.77	5.81–20.60	0.000
Hypertension	3.32	3.56	1.43–8.85	0.006
Low level of physical activity	2.09	2.86	1.97–8.42	0.046
Decreased handgrip strength	2.14	1.06	1.03–1.12	0.045
FEV_1_ (L) < 80%	3.22	2.33	1.14–4.76	0.022
Body Mass Index > 30 kg/m^2^	2.96	2.78	1.09–7.11	0.032
Pseudo R^2^ = 0.407. Hosmer–Lemeshow Chi^2^ 147.83 (*p* = 0.973). AUC = 0.902				

OR: odds ratio; CI: confidence interval.

## Data Availability

The original contributions presented in the study are included in the article, and further inquiries can be directed at the corresponding authors.

## Supplementary Materials

The following supporting information can be downloaded at: https://www.mdpi.com/article/10.3390/jcm15051868/s1, Table S1: The STROBE reporting checklist; Table S2: Demographic and clinical baseline data of the living and dead subjects; Table S3: Total vaccine doses administered; Table S4: Types of vaccines administered to the COVID and non-COVID population; Table S5: Pre- and post-pulmonary function parameters in COVID-19 and control participants; Table S6: Pre-COVID-19 pulmonary function parameters in living and dead subjects; Table S7: Predicted values of pre-COVID-19 pulmonary function tests in living and dead subjects.

## Abbreviations

The following abbreviations are used in this manuscript:

COVID-19	Coronavirus Disease 2019
LF	Lung Function
PURE	Prospective Urban Rural Epidemiology
FEV_1_	Forced Expiratory Volume in 1 Second
FVC	Forced Vital Capacity
PFT	Pulmonary Function Test
BMI	Body Mass Index
PEF	Peak Expiratory Flow
GLI	Global Lung Initiative
HS	Handgrip Strength
WHR	Waist-to-Hip Ratio

## References

[1] Zhu N., Zhang D., Wang W., Li X., Yang B., Song J., Zhao X., Huang B., Shi W., Lu R. (2020). A Novel Coronavirus from Patients with Pneumonia in China, 2019. N. Engl. J. Med..

[2] Hui D.S., Joynt G.M., Wong K.T., Gomersall C.D., Li T.S., Antonio G., Ko F.W., Chan M.C., Chan D.P., Tong M.W. (2005). Impact of severe acute respiratory syndrome on pulmonary function, functional capacity and quality of life in a cohort of survivors. Thorax.

[3] Ong K.C., Ng A.W., Lee L.S., Kaw G., Kwek S.K., Leow M.K., Earnest A. (2005). 1-year pulmonary function and health status in survivors of severe acute respiratory syndrome. Chest.

[4] Afsin E., Demirkol M.E. (2022). Post-COVID pulmonary function test evaluation. Turk. Thorac. J..

[5] Shi H., Han X., Jiang N., Cao Y., Alwalid O., Gu J., Fan Y., Zheng C. (2020). Radiological findings from 81 patients with COVID-19 pneumonia in Wuhan, China: A descriptive study. Lancet Infect. Dis..

[6] Sodhi M.K., Aggarwal D., Puri S., Saini V. (2023). Persistent respiratory symptoms and lung function abnormalities in recovered patients of COVID-19. Lung India.

[7] Suppini N., Fira-Mladinescu O., Traila D., Motofelea A.C., Marc M.S., Manolescu D., Vastag E., Maganti R.K., Oancea C. (2023). Longitudinal analysis of pulmonary function impairment one year post-COVID-19: A single-center study. J. Pers. Med..

[8] Zhang H., Li X., Huang L., Gu X., Wang Y., Liu M., Liu Z., Zhang X., Yu Z., Wang Y. (2022). Lung-function trajectories in COVID-19 survivors after discharge: A two-year longitudinal cohort study. EClinicalMedicine.

[9] Huang Y., Tan C., Wu J., Chen M., Wang Z., Luo L., Zhou X., Liu X., Huang X., Yuan S. (2020). Impact of coronavirus disease 2019 on pulmonary function in early convalescence phase. Respir. Res..

[10] Bemba E.L.P., Okombi F.O., Moyikoua R., Bopaka R.G., Koumeka P.P., Ossale-Abacka K.B., Moukassa D., Mboussa J. (2024). Post-COVID-19 pneumonia: Long-term radiographic and spirometric outcomes. J. Pan Afr. Thorac. Soc..

[11] Tabernero Huguet E., Urrutia-Gajarte A., Ruíz-Uturriaga L.A., Serrano-Fernández L., Marina-Malanda N., Iriberri-Pascual M., Zalazain-Jorge R. (2021). Alteración funcional pulmonar en el seguimiento precoz de pacientes con neumonía por COVID-19. Arch. Bronconeumol..

[12] Mogensen I., Hallberg J., Björkander S., Du L., Zuo F., Hammarström L., Pan-Hammarström Q., Ekström S., Georgelis A., Palmberg L. (2022). Lung functions before and after COVID-19 in young adults: A population-based study. J. Allergy Clin. Immunol. Glob..

[13] Taib R.R., Kozlov Y., Ekshtein A., Gordon B., Wand O., Ben-Ari O. (2025). A comparison of pulmonary function pre and post mild SARS-CoV-2 infection among healthy adults. BMC Pulm. Med..

[14] Torres-Castro R., Vasconcello-Castillo L., Alsina-Restoy X., Solis-Navarro L., Burgos F., Puppo H., Vilaró J. (2021). Respiratory function in patient’s post-infection by COVID-19: A systematic review and meta-analysis. Pulmonology.

[15] Udwadia Z.F., Koul P.A., Richeldi L. (2021). Post-COVID lung fibrosis: The tsunami that will follow the earthquake. Lung India.

[16] Cornelissen M.E., Leliveld A., Baalbaki N., Gach D., van der Lee I., Nossent E.J., Bloemsma L.D., Maitland-van der Zee A.H. (2024). Pulmonary function 3–6 months after acute COVID-19: A systematic review and multicentre cohort study. Heliyon.

[17] Ball L., Barisione E., Mastracci L., Campora M., Costa D., Robba C., Battaglini D., Brunetti I., Fiasella S., Giacobbe D.R. (2021). Extension of collagen deposition in COVID-19 postmortem lung samples and computed tomography analysis findings. Int. J. Mol. Sci..

[18] Barisione E., Grillo F., Ball L., Bianchi R., Grosso M., Morbini P., Mastracci L., Fiocca R., Patroniti N., De Lucia A. (2021). Fibrotic progression and radiologic correlation in matched lung samples from COVID-19 postmortems. Virchows Arch..

[19] Mara G., Nini G., Cotoraci C. (2025). Impact of pulmonary comorbidities on COVID-19: Acute and long-term evaluations. J. Clin. Med..

[20] Teo K., Chow C.K., Vaz M., Rangarajan S., Yusuf S. (2009). The Prospective Urban Rural Epidemiology (PURE) study: Examining the impact of societal influences on chronic noncommunicable diseases in low-, middle-, and high-income countries. Am. Heart J..

[21] Craig C.L., Marshall A.L., Sjostrom M., Bauman A.E., Booth M.L., Ainsworth B.E., Pratt M., Ekelund U., Yngve A., Sallis J.F. (2003). International physical activity questionnaire: 12-country reliability and validity. Med. Sci. Sports Exerc..

[22] Lear S.A., Hu W., Rangarajan S., Gasevic D., Leong D., Iqbal R., Casanova A., Swaminathan S., Anjana R.M., Kumar R. (2017). The effect of physical activity on mortality and cardiovascular disease in 130,000 people from 17 high-income, middle-income, and low-income countries. Lancet.

[23] Graham B.L., Steenbruggen I., Miller M.R., Barjaktarevic I.Z., Cooper B.G., Hall G.L., Hallstrand T.S., Kaminsky D.A., McCarthy K., McCormack M.C. (2019). Standardization of Spirometry 2019 Update. An Official American Thoracic Society and European Respiratory Society Technical Statement. Am. J. Respir. Crit. Care Med..

[24] Duong M., Islam S., Rangarajan S., Leong D., Kurmi O., Teo K., Killian K., Dagenais G., Lear S., Yusuf S. (2013). Global differences in lung function by region (PURE): An international, community-based prospective study. Lancet Respir. Med..

[25] Duong M., Rangarajan S., Zaman M., Nasir N.M., Seron P., Yeates K., Yusufali A.M., Khatib R., Tse L.A., Wang C. (2022). Differences and agreement between two portable hand-held spirometers across diverse community-based populations in the PURE study. PLoS Glob. Public Health.

[26] Bowerman C., Bhakta N.R., Brazzale D., Cooper B.R., Cooper J., Gochicoa-Rangel L., Hall G.L., Kulkarni T., Miller M.R., Pellegrino R. (2023). A racially neutral approach to the interpretation of lung function measurements. Am. J. Respir. Crit. Care Med..

[27] Leong D.P., Teo K.K., Rangarajan S., Kutty V.R., Lanas F., Hui C., Quanyong X., Zhenzhen Q., Jinhua T., Noorhassim I. (2016). Reference ranges of handgrip strength from 125,462 healthy adults in 21 countries. J. Cachexia Sarcopenia Muscle.

[28] Cohen J. (2013). Statistical Power Analysis for the Behavioral Sciences.

[29] von Elm E., Altman D.G., Egger M., Pocock S.J., Gøtzsche P.C., Vandenbroucke J.P. (2007). The Strengthening the Reporting of Observational Studies in Epidemiology (STROBE) statement: Guidelines for reporting observational studies. Lancet.

[30] Iversen K.K., Afzal S., Ahlström M.G., Nordestgaard B.G., Schneider U.V., Nielsen L., Kofoed K., Benfield T., Ronit A. (2022). Lung function decline in relation to COVID-19 in the general population: A matched cohort study with prepandemic assessment in lung function. J. Infect. Dis..

[31] Lewis K.L., Helgeson S.A., Tatari M.M., Mallea J.M., Baig H.Z., Patel N.M. (2021). COVID-19 and the effects on pulmonary function following infection: A retrospective analysis. EClinicalMedicine.

[32] Bostancı Ö., Karaduman E., Çolak Y., Yılmaz A.K., Kabadayı M., Bilgiç S. (2023). Respiratory muscle strength and pulmonary function in unvaccinated athletes before and after COVID-19 infection: A prospective cohort study. Respir. Physiol. Neurobiol..

[33] Ippoliti L., Coppeta L., Somma G., Bizzarro G., Borelli F., Crispino T., Ferrari C., Iannuzzi I., Mazza A., Paolino A. (2023). Pulmonary function assessment after COVID-19 in vaccinated healthcare workers. J. Occup. Med. Toxicol..

[34] Smith C.B., Golden C.A., Kanner R.E., Klauber M.R., Renzetti A.D. (1980). Effect of viral infections on pulmonary function in patients with chronic obstructive pulmonary disease. J. Infect. Dis..

[35] Johanson W.G., Pierce A.K., Sanford J.P. (1969). Pulmonary function in uncomplicated influenza. Am. Rev. Respir. Dis..

[36] Mo X., Jian W., Su Z., Chen M., Peng H., Peng P., Lei C., Chen R., Zhong N., Li S. (2020). Abnormal pulmonary function in COVID-19 patients at time of hospital discharge. Eur. Respir. J..

[37] Shah D.P., Thaweethai T., Karlson E.W., Bonilla H., Horne B.D., Mullington J.M., Wisnivesky J.P., Hornig M., Shinnick D.J., Klein J.D. (2025). Sex differences in long COVID. JAMA Netw. Open.

[38] van Willigen H.D.G., Wynberg E., Verveen A., Dijkstra M., Verkaik B.J., Figaroa O.J.A., de Jong M.C., van der Veen A.L.I.P., Makowska A., Koedoot N. (2023). One-fourth of COVID-19 patients have an impaired pulmonary function after 12 months of disease onset. PLoS ONE.

[39] Rosas I.O., Benitez A., McKinnell J.A., Shah R., Waters M., Hunter B.D., Jeanfreau R., Tsai L., Neighbors M., Trzaskoma B. (2025). Long-term clinical outcomes of adults hospitalized for COVID-19 pneumonia. Emerg. Infect. Dis..

[40] Chai C.S., Bin Ibrahim M.A., Binti Azhar N.A., Binti Roslan Z., Binti Harun R., Krishnabahawan S.L., Karthigayan A.A.P., Kadir R.F.B.A., Johari B.B., Ng D.-L. (2024). Post-discharge spirometry evaluation in patients recovering from moderate-to-critical COVID-19: A cross-sectional study. Sci. Rep..

[41] Larsson A.C., Palstam A., Ashman Kröönström L., Sunnerhagen K.S., Persson H.C. (2025). Factors associated with aspects of functioning one year after hospitalization due to COVID-19. Clin. Rehabil..

[42] Pini L., Guerini M., Giordani J., Levi G., Latronico N., Piva S., Peli E., Benoni R., Pini A., Zucchi G. (2025). 24-month assessment of respiratory function in patients hospitalized for severe SARS-CoV-2 pneumonia: A follow-up study. Intern. Emerg. Med..

[43] Fumagalli A., Misuraca C., Bianchi A., Borsa N., Limonta S., Maggiolini S., Bonardi D.R., Corsonello A., Di Rosa M., Soraci L. (2022). Long-term changes in pulmonary function among patients surviving COVID-19 pneumonia. Infection.

[44] Pietranis K.A., Izdebska W.M., Kuryliszyn-Moskal A., Dakowicz A., Ciołkiewicz M., Kaniewska K., Dzięcioł-Anikiej Z., Wojciuk M. (2024). Effects of pulmonary rehabilitation on respiratory function and thickness of the diaphragm in patients with post-COVID-19 syndrome: A randomized clinical trial. J. Clin. Med..

[45] Harris E. (2023). Study: Waist-to-hip ratio might predict mortality better than BMI. JAMA.

[46] Corsi A., Caroli A., Bonaffini P.A., Conti C., Arrigoni A., Mercanzin E., Imeri G., Anelli M., Balbi M., Pace M. (2022). Structural and functional pulmonary assessment in severe COVID-19 survivors at 12 months after discharge. Tomography.

[47] do Amaral C.M.S.S.B., Goulart C.d.L., Silva B.M., Valente J., Rezende A.G., Fernandes E., Cubas-Vega N., Borba M.G.S., Sampaio V., Monteiro W. (2024). Low handgrip strength is associated with worse functional outcomes in long COVID. Sci. Rep..

[48] Nagel C., Olschewski H., Sorichter S., Uezgoer G., Diehm C., Huppert P., Iber T., Herth F., Harutyunova S., Marra A.M. (2022). Impairment of inspiratory muscle function after COVID-19. Respiration.

[49] Hockele L.F., Affonso J.V.S., Rossi D., Eibel B. (2022). Pulmonary and functional rehabilitation improves functional capacity, pulmonary function and respiratory muscle strength in post-COVID-19 patients: Pilot clinical trial. Int. J. Environ. Res. Public Health.

[50] Ganaza-Domingues K.L.T., Ramos-Milaré Á.C.F.H., Lera-Nonose D.S.S.L., Brustolin A.Á., de Oliveira L.F., Rosa J.S., Otofuji Inada A.Y., Dias Leme A.L., Pinel B.I., Perina B.S. (2025). Effect of comorbidities on mortality of patients with COVID-19: A systematic review of reviews and meta-analysis. Rev. Med. Virol..

[51] Zhou G., Dael N., Verweij S., Balafas S., Mubarik S., Oude Rengerink K., Pasmooij A.M.G., van Baarle D., Mol P.G., de Bock G.H. (2025). Effectiveness of COVID-19 vaccines against SARS-CoV-2 infection and severe outcomes in adults: A systematic review and meta-analysis of European studies published up to 22 January 2024. Eur. Respir. Rev..

[52] Hamrouni M., Roberts M.J., Bishop N.C. (2023). High grip strength attenuates risk of severe COVID-19 in males but not females with obesity: A short-communication of prospective findings from UK Biobank. Obes. Res. Clin. Pract..

[53] Rostamzadeh S., Allafasghari A., Allafasghari A., Abouhossein A. (2024). Handgrip strength as a prognostic factor for COVID-19 mortality among older adult patients admitted to the intensive care unit (ICU): A comparison Alpha (B.1.1.7) and Delta (B.1.617.2) variants. Sci. Rep..

[54] Pucci G., D’abbondanza M., Curcio R., Alcidi R., Campanella T., Chiatti L., Gandolfo V., Veca V., Casarola G., Leone M.C. (2022). Handgrip strength is associated with adverse outcomes in patients hospitalized for COVID-19-associated pneumonia. Intern. Emerg. Med..

[55] Pinto F.C.S., Andrade M.F., Gatti da Silva G.H., Faiad J.Z., Barrére A.P.N., Gonçalves R.d.C., de Castro G.S., Seelaender M. (2022). Function over mass: A meta-analysis on the importance of skeletal muscle quality in COVID-19 patients. Front. Nutr..

