# Supplementary material for: Impact of SARS-CoV-2 Infection on Pulmonary Function in the PURE-Colombia Cohort: A Comparative Analysis with Pre-COVID Values and Non-COVID-19 Controls
Source: J Clin Med. 2026 Feb 28;15(5):1868. doi: 10.3390/jcm15051868 (PMC12985542; doi:10.3390/jcm15051868)
Supplement: Supplementary file 1 [file jcm-15-01868-s001.zip › jcm-4129866-supplementary.pdf]

IMPACT OF SARS-COV-2 INFECTION ON PULMONARY FUNCTION IN THE PURE COLOMBIA COHORT: A COMPARATIVE ANALYSIS WITH PRE-COVID VALUES AND NON COVID 19 CONTROLS

Supplementary material

	Page
**Table S1.** The STROBE reporting checklist	2
**Table S2.** Demographic and clinical baseline data of the living and dead subjects.	5
**Table S3.** Total vaccine doses administered.	6
**Table S4.** Types of vaccines administered to the COVID and non-COVID population.	7
**Table S5.** Pre- and post-pulmonary function parameters in COVID-19 and control participants.	8
**Table S6.** Pre-COVID-19 pulmonary function parameters in living and dead subjects.	9
**Table S7.** Predicted values of pre-COVID-19 pulmonary function tests in living and dead subjects	10

Table S1. The STROBE reporting checklist

	Item Description	Location (or reason for not reporting)
**Title and abstract**		Page 1
1a. Indicate the study's design	Indicate the study's design with a commonly used term in the title or the abstract.	Page 2
1b. Abstract	Provide in the abstract an informative and balanced summary of what was done and what was found.	Page 2
**Introduction**		
2. Background / rationale	Explain the scientific background and rationale for the investigation being reported.	Introduction; paragraph 1 & 2
3. Objectives	State specific objectives, including any prespecified hypotheses.	Introduction; paragraph 2 & 3
**Methods**		
4. Study design	Present key elements of study design early in the paper.	Methods; paragraph 1
5. Setting	Describe the setting, locations, and relevant dates, including periods of recruitment, exposure, follow-up, and data collection.	Methods; paragraph 1 & 2
6a. Eligibility criteria	**Cohort study:** Give the eligibility criteria, and the sources and methods of selection of participants. Describe methods of follow-up. **Case-control study:** Give the eligibility criteria, and the sources and methods of case ascertainment and control selection. Give the rationale for the choice of cases and controls. **Cross-sectional study:** Give the eligibility criteria, and the sources and methods of selection of participants.	Methods; paragraph 1 & 2
6b. Matching criteria	**Cohort study:** For matched studies, give matching criteria and number of exposed and unexposed. **Case-control study:** For matched studies, give matching criteria and the number of controls per case.	Not applicable
7. Variables	Clearly define all outcomes, exposures, predictors, potential confounders, and effect modifiers. Give diagnostic criteria, if applicable.	Methods; paragraph 3
8. Data sources / measurement	For each variable of interest give sources of data and details of methods of assessment (measurement). Describe comparability of assessment methods if there is more than one group.	Methods; paragraph 3 – 6
9. Bias	Describe any efforts to address potential sources of bias.	
10. Study size	Explain how the study size was arrived at.	Methods; paragraph 1 & 2
11. Quantitative variables	Explain how quantitative variables were handled in the analyses. If applicable, describe which groupings were chosen, and why.	Methods; paragraph 3
12a. Statistical methods	Describe all statistical methods, including those used to control for confounding.	Methods; paragraph 7 – 11
12b. Statistical methods – subgroups and interactions	Describe any methods used to examine subgroups and interactions.	Methods; paragraph 7 – 11
12c. Statistical methods – missing data	Explain how missing data were addressed.	Methods; paragraph 7 & 8

	Item Description	Location (or reason for not reporting)
12di. Statistical methods – loss to follow-up	**Cohort study:** If applicable, describe how loss to follow-up was addressed.	
12dii. Statistical methods – matching cases and controls	**Case-control study:** If applicable, explain how matching of cases and controls was addressed.	
12diii. Statistical methods – sampling strategy	**Cross-sectional study:** If applicable, describe analytical methods taking account of sampling strategy.	Methods; paragraph 1 & 2
12e. Statistical methods – sensitivity analyses	Describe any sensitivity analyses.	Methods; paragraph 10 & 11
**Results**		
13a. Participant numbers	Report the numbers of individuals at each stage of the study—e.g., numbers potentially eligible, examined for eligibility, confirmed eligible, included in the study, completing follow-up, and analysed; Consider use of a flow diagram.	Results; paragraph 1; Figure 1
13b. Participants – non-participation	Give reasons for non-participation at each stage.	Results; paragraph 1
13c. Participants – flow diagram	Consider use of a flow diagram.	Figure 1
14a. Descriptive data – participant characteristics	Give characteristics of study participants (e.g., demographic, clinical, social) and information on exposures and potential confounders. Present the information in a table.	Results; paragraph 1 & 2; Table 1
14b. Descriptive data – missing data	Indicate the number of participants with missing data for each variable of interest.	Results; paragraph 3; Figure 1
14c. Descriptive data – follow-up time	**Cohort study:** Summarise follow-up time—e.g., average and total amount.	Results; paragraph 3 – 7; Figure 2 & 3
15. Outcome data	**Cohort study:** Report numbers of outcome events or summary measures over time. **Case-control study:** Report numbers in each exposure category, or summary measures of exposure. **Cross-sectional study:** Report numbers of outcome events or summary measures.	Results; paragraph 3 – 7
16a. Main results	Give unadjusted estimates and, if applicable, confounder-adjusted estimates and their precision (e.g., 95% confidence intervals). Make clear which confounders were adjusted for and why they were included.	Results; paragraph 8 – 10; Table 2 & 3
16b. Main results – category boundaries	Report category boundaries when continuous variables were categorised.	Results; paragraph 5 & 6
16c. Main results – risk	If relevant, consider translating estimates of relative risk into absolute risk for a meaningful time period.	Results; paragraph 10
17. Other analyses	Report other analyses done—e.g., analyses of subgroups and interactions, and sensitivity analyses.	Results; paragraph 7; Figure 4
**Discussion**		
18. Key results	Summarise key results with reference to study objectives.	Discussion; paragraph 1 & 2
19. Limitations	Discuss limitations of the study, taking into account sources of potential bias or imprecision. Discuss both direction and magnitude of any potential bias.	Discussion; paragraph 12
20. Interpretation	Give a cautious overall interpretation considering objectives, limitations, multiplicity of analyses, results from similar studies, and other relevant evidence.	Discussion; paragraph 3 – 11

	Item Description	Location (or reason for not reporting)
21. Generalisability	Discuss the generalisability (external validity) of the study results.	Discussion; paragraph 10 & 11
**Other information**		
22. Funding	Give the source of funding and the role of the funders for the present study and, if applicable, for the original study on which the present article is based.	Funding and Acknowledgements

Source: Elm E von, Altman DG, Egger M, Pocock SJ, Gøtzsche PC, Vandenbroucke JP, et al. The STROBE reporting checklist. In: Harwood J, Albury C, Beyer J de, Schlüssel M, Collins G, editors. The EQUATOR network reporting guideline platform [Internet]. The UK EQUATOR Centre; 2025. Available from: https:/resources.equator-network.org/reporting-guidelines/strobe/strobe-checklist.docx

Table S2. Demographic and clinical baseline data of the living and dead subjects.

Variable		Living Subjects (n=149)		Dead Subjects (n=51)		p-value*
		n	%	n	%	
**Gender**	Female	95	63.8	22	43.1	0.015
	Male	54	36.2	29	56.9	
**Age**	< 60 years	65	43.6	5	9.8	0.000
	≥ 60 years	84	56.4	46	90.2	
**Educational level**	Low[1]	78	52.3	27	52.9	0.928
	Middle[2]	34	22.8	18	35.3	
	High[3]	37	24.8	6	11.8	
**Health insurance[4]**	Contributory	88	59.1	29	43.1	0.912
	Subsidized[4]	61	40.9	22	56.9	
**Place of residence**	Urban	70	47.0	24	46.0	0.878
	Rural	79	53.0	27	54.0	
**Body mass index (BMI)** (kg/m^2)	Normal: 18.5-24.9	51	34.2	17	33.3	0.956
	Overweight: 25-29.9	69	46.3	20	39.2	
	Obesity: ≥ 30	29	19.5	14	27.5	
**Background**	Smoking	53	35.6	32	62.8	0.001
	COPD / asthma	6	4.0	2	3.9	0.703
	Hypertension	43	28.9	33	64.7	0.000
	Diabetes mellitus	15	10.1	9	17.6	0.234
**Blood groups**	O blood type	103	69.2	19	37.3	0.001
	Non-O blood types	46	30.8	32	62.7	
**Physical activity level** (min/week)	Mild <150	24	16.1	14	27.5	0.115
	Moderate 150 – 750	58	38.9	16	31.4	
	High > 750	67	45.0	19	37.3	
**Waist-to-hip index**	Normal[5]	78	52.3	18	35.3	0.052
	High	71	47.7	33	64.7	
**Handgrip strength** (kg)	Normal	110	73.8	26	51.0	0.004
	Decreased [6]	39	26.2	25	49.0	
**Vaccination status prior to COVID-19**	Complete scheme [7]	31	20.8	7	13.7	0.000
	No vaccine dose	100	67.1	37	72.6	
	Incomplete scheme	18	12.1	7	7.8	
**Laboratories (mg/dL)** (mean ± SD)	Creatinine	0.99±0.23		0.93±0.17		0.050
	Non-HDL cholesterol	154±43.1		146.7±50.1		0.355
	Triglycerides	169±84.0		228.5±182.1		0.028

[1]Low: no education, primary education, lower secondary education; [2]Middle: complete secondary education; [3]High: technician, bachelor, master, doctoral or equivalent; [4]Colombia´s compulsory health insurance regime. [5] Women: 0.71-0.85 cm; Men: 0.78-0.94. [6] Women: <19 kg; Men: <32 kg. [7] One dose of Janssen vaccine and two doses of the other vaccines. * Chi^2 test for difference proportions and T Student test for difference proportions.

Table S3. Total vaccine doses administered.

VACCINE	NO COVID* (n=402)		COVID-19 Not hospitalized (n=127)		Hospitalized (n=22)		Dead (n= 51)	
	**n**	**%**	**n**	**%**	**n**	**%**	**n**	**%**
No vaccine	286	71.1%	87	68.5%	13	59.0%	37	72.60%
Incomplete vaccination	21	5.2%	13	10,2%	4	18.20%	7	13.70%
Complete vaccination	95	23.6%	27	21.3%	5	22.80%	7	13.70%

* Vaccination recorded at the time of performing spirometry

Table S4. Types of vaccines administered to the COVID and non-COVID population.

VACCINE GROUP	VACCINE	NO COVID (n=402)	COVID-19		
			Not hospitalized (n= 127)	Hospitalized (n= 22)	Dead (n= 51)
**ARNm**	Modern	2	6	3	0
	Pfizer	103	40	5	2
**Viral vector**	Aztrazeneca	64	10	2	3
	JANSSEN	8	0	0	0
**Inactivacted virus**	Sinovac	40	18	6	18

Table S5. Pre- and post-LF parameters in COVID-19 and control participants.

Variable	Basal				Post-COVID or Follow-up*				Diff.	T o Z Statistic	Size of e effect**	*p*-value
	$\overline{X}$	SD	Median	IQR	$\overline{X}$	SD	Median	IQR	$\overline{X}$			
**COVID-19 (n=149)**												
**Outpatient (n=127)**												
**FEV$_1$ (L)**	2.43	0.73	2.36	0.78 - 4.13	2.41	0.65	2.18	0.99 – 4.34	-0.02	0.728	0.031	0.233
**FVC (L)**	2.61	0.88	2.63	0.59 – 4.39	2.60	0.76	2.42	0.90 – 4.77	-0.01	0.175	0.015	0.437
**FEV$_1$/FVC (%)**	0.97	0.22	0.94	0.66 – 1.98	0.96	0.16	0.88	0.37 – 1.21	-0.01	0.128	0.07	0.234
**PEF (L/s)**	363	137	332	90 - 771	354	133	302	81 - 718	-9.34	1.283	0.068	0.109
**Hospitalized (n=22)**												
**FEV$_1$ (L)**	2.84	0.78	2.73	1.14 – 3.99	2.34	0.75	2.32	0.99 – 3.71	-0.50	3.247	0.656	**0.002**
**FVC (L)**	3.01	0.85	2.96	0.96 – 4.17	2.53	0.89	2.37	0.23 – 4.15	-0.48	2.686	0.549	**0.006**
**FEV$_1$/FVC (%)**	0.95	0.11	0.95	0.80 – 1.22	1.06	0.73	0.9	0.73 – 4.30	0.11	0.704	0.109	0.755
**PEF (L/s)**	399	171	346	123 - 703	328.7	152	334	23 - 574	-70.6	3.338	0.434	**0.001**
**Non-COVID-19 (n=402)**												
**FEV$_1$ (L)**	2.29	0.70	2.27	1.83-2.70	2.20	0.72	2.13	1.72-2.65	-0.09	9.175	0.193	0.058
**FVC (L)**	2.64	0.88	2.64	2.17-3.18	2.55	0.83	2.44	2.00-2.98	-0.09	5.705	0.113	0.056
**FEV$_1$/FVC (%)**	0.91		0.89	0.81-0.96	0.88	0.23	0.88	0.82-0.92	-0.03	1.177	0.069	0.239
**PEF (L/s)**	304	117	293	219-374	290	125	288	202-365	-14	5.000	0.164	0.057

* Post COVID-19 LF for subjects with SARS-CoV-2 infection and follow-up LF measurement for controls.

**For the Student paired t-test (t statistic), the effect size is expressed by Cohen´s d. For the Wilcoxon test (z statistic), the effect size is expressed by de paired rank biserial correlation.

$\overline{X}$: average; SD: Standard Deviation; IQR: Interquartile Range; FEV1: Forced Expiratory Volume in the first second; FVC: Forced Vital Capacity; PEF: Peak Expiratory Flow.

Table S6. Pre-COVID-19 pulmonary function parameters in living and dead subjects.

Variable	Living Subjects (n=149)				Dead Subjects (n=51)				p-value*
	$\overline{X}$	SD	Median	IQR	$\overline{X}$	SD	Median	IQR	
**FEV_1 (L)**	2.49	0.75	2.44	(0.78 – 4.13)	2.02	0.86	1.95	(1.53 - 2.41)	0.000
**FVC (L/s)**	2.67	0.88	2.69	(0.59 – 4.39)	2.22	0.95	2.11	(1.76 – 2.74)	0.003
**FEV_1/FVC (%)**	0.95	0.11	0.94	(0.66 – 1.98)	0.96	0.25	0.95	(0.91 – 0.99)	0.783
**PEF (L/s)**	368.99	142.60	333.00	(90 - 771)	377.80	156.74	272.0	(168.0 – 391.0)	0.724

*T Student test for difference means ($\overline{X}$).

Table S7. Predicted values of pre-COVID-19 pulmonary function tests in living and dead subjects.

Variable (% Predicted)		Living Subjects (n=149)		Dead Subjects (n=51)		p-value*
		n	%	n	%	
**FEV_1 (L)**	<70%	18	12.1	10	19.6	0.269
	≥70%	131	87.9	41	80.4	
**FVC (L/s)**	<80%	44	29.5	25	49.0	0.018
	≥80%	105	70.5	26	51.0	
**FEV_1/FVC (%)**	<70%	0	0.0	0	0.0	1.000
	≥70%	149	100.0	51	100.0	
**PEF (L/s)**	<80%	83	55.7	35	68.6	0.145
	≥80%	66	44.3	16	31.4	

*Chi^2 test for difference proportions.

FEV_1: Forced Expiratory Volume in the first second; FVC: Forced Vital Capacity; PEF: Peak Expiratory Flow.
